# Adipose Extracellular Vesicles in Intercellular and Inter-Organ Crosstalk in Metabolic Health and Diseases

**DOI:** 10.3389/fimmu.2021.608680

**Published:** 2021-02-25

**Authors:** Zhe Huang, Aimin Xu

**Affiliations:** ^1^The State Key Laboratory of Pharmaceutical Biotechnology, The University of Hong Kong, Hong Kong, China; ^2^Department of Medicine, The University of Hong Kong, Hong Kong, China; ^3^Department of Pharmacology and Pharmacy, The University of Hong Kong, Hong Kong, China

**Keywords:** exosome, microRNA, adipose tissue macrophage, metabolic homeostasis, inflammation, cell-cell communication

## Abstract

Adipose tissue (AT) is a highly heterogeneous and dynamic organ that plays important roles in regulating energy metabolism and insulin sensitivity. In addition to its classical roles in nutrient sensing and energy storage/dissipation, AT secretes a large number of bioactive molecules (termed adipokines) participating in immune responses and metabolic regulation through their paracrine and/or endocrine actions. Adipose-derived extracellular vesicles (ADEVs), including exosomes, microvesicles (MVs), and apoptotic bodies, have recently emerged as a novel class of signal messengers, mediating intercellular communications and inter-organ crosstalk. In AT, ADEVs derived from adipocytes, immune cells, mesenchymal stem cells, endothelial cells are actively involved in modulation of immune microenvironment, adipogenesis, browing of white adipose tissue, adipokine release and tissue remodeling. Furthermore, ADEVs exert their metabolic actions in distal organs (such as liver, skeletal muscle, pancreas and brain) by sending genetic information (mainly in the form of microRNAs) to their target cells for regulation of gene expression. Here, we provide an updated summary on the nature and composition of ADEVs, and their pathophysiological functions in regulating immune responses, whole-body insulin sensitivity and metabolism. Furthermore, we highlight the latest clinical evidence supporting aberrant production and/or function of ADEVs as a contributor to obesity-related chronic inflammation and metabolic complications and discuss the opportunities and challenges in developing novel therapies by targeting ADEVs.

## Introduction

Obesity, characterized by excessive accumulation of adipose tissue (fat), is a highly complex multifaceted chronic disease and one of the major risk factors for a cluster of cardio-metabolic diseases, including type 2 diabetes (T2D), dyslipidemia, non-alcoholic fatty liver disease (NAFLD), hypertension, coronary heart disease and stroke (1). Furthermore, obesity-related complications are the most commonly reported underlying conditions that predispose individuals with viral infections, including the current coronavirus disease (COVID-19) to severe outcomes (2).

Adipose tissue is a complex metabolic organ with profound effects on the regulation of systemic metabolism and maintenance of energy homeostasis. In addition to its classical role in nutrient handling, including energy storage in the form of triglycerides during feeding and releasing of free fatty acids (FFAs) during fasting, adipose tissue also serves as an active endocrine organ secreting a variety of adipokines, which are bioactive peptides including cytokines, peptide hormones and enzymes acting in an autocrine, paracrine or endocrine manner to regulate energy metabolism, immune responses and cardiovascular homeostasis. Adipokines have been shown to modulate adipogenesis, adipocyte metabolism, and immune cell infiltration locally within adipose tissue. Additionally, they can exert their biological effects in distal organs to maintain systemic energy homeostasis and insulin sensitivity (3, 4). However, during obesity, adipose tissue undergoes unhealthy expansion leading to numerous detrimental consequences, including dysregulated secretion of adipokines, hypoxia, cell death and altered immune microenvironment which give rise to adipose inflammation (5). Unresolved chronic inflammation in adipose tissue is a major contributor to systemic low-grade inflammation and has been reported as a culprit in obesity-related comorbidities. The first evidence for adipose inflammation at the interface between obesity and metabolic dysregulation was provided by studies demonstrating increased production and secretion of the inflammatory cytokine tumor necrosis factor alpha (TNFα) from adipose tissues in obese rodents and human subjects (6, 7). Neutralization of TNFα in obese rats counteracted diet-induced insulin resistance and glucose intolerance (7). Subsequent studies reported that selective inactivation of pro-inflammatory signaling pathways in adipose tissue by inhibition of key signaling molecules, including c-Jun N-terminal kinase (JNK) and nuclear factor-κB (NF-κB) disrupted the link between obesity and metabolic dysregulation (8–10).

In addition to the classical polypeptide adipokines and cytokines, various types of cells in adipose tissue also produce and release extracellular vesicles (EVs) including exosomes with a diameter of 30–100 nm originated from cytoplasmic multivesicular bodies that fuse with the plasma membrane and microvesicles (MVs) that are 100–1000 nm in diameter and released directly from the plasma membrane into the extracellular space. Both types of such adipose-derived extracellular vesicles (ADEVs) are similar to the original cells in composition, transporting bioactive molecules, including proteins, lipids, and nucleic acids to their target cells within adipose tissue or in distant organs, therefore mediating intercellular and interorgan crosstalk. A growing body of evidence suggest that ADEVs play important roles in the regulation of metabolic inflammation, energy metabolism and insulin sensitivity (11–15). Altered abundance or content of ADEVs may be causally linked to obesity-related metabolic complications.

In this review, we summarize the nature and compositions of ADEVs derived from different cellular origins in adipose tissue and their roles as local and/or distal signaling mediators in regulating metabolic homeostasis. Furthermore, we highlight the latest evidence for the clinical association of aberrant production and/or functions of ADEVs with various obesity-related metabolic disorders, and discuss the therapeutic potentials of targeting ADEVs for the treatment of obesity-related metabolic complications and challenges in this field.

## Adipose Tissue In Health and Metabolic Diseases

### Heterogeneity of Adipose Tissue

Adipose tissue in mammals is categorized into two main types, white adipose tissue (WAT) and brown adipose tissue (BAT). WAT mainly consists of white adipocytes, which contain a single large lipid droplet (referred to as unilocular lipid structure) and few mitochondria, thus is a primary site for energy storage. White adipocytes are highly responsive to hormones such as insulin to take up and store nutrients in the form of triglycerides after food ingestion. They also respond to biogenic amines such as catecholamines to supply energy in the forms of FFAs and glycerol during nutrient deprivation (5). WAT is distributed throughout the body. Main depots include subcutaneous adipose tissue (SAT), which is beneath the skin storing more than 80% of total fat in the body and is mainly located in the abdominal and gluteofemoral regions in humans or between the scapulae and in the inguinal region spreading from the dorsolumbar to the gluteal region in rodents, and visceral adipose tissue (VAT), which stores 5–20% of total body fat and is associated with internal organs mainly in perigonadal, mesenteric, retroperitoneal, epicardial and periadventitial regions in rodents and humans (16, 17). In addition, there are also small adipose depots including epicardial and intermuscular adipose tissue with specialized functions related to cardiovascular system or skeletal muscle (17). While the major function of SAT is to store excess energy in response to energy surplus and is therefore considered as beneficial, VAT is more closely linked to adverse metabolic profile and inflammation in obese subjects (18, 19).

BAT mainly consists of brown adipocytes with multilocular lipid droplets and a large number of highly oxidative, naturally uncoupled mitochondria, and is important for the regulation of body temperature through non-shivering thermogenesis. The thermogenic capacity of brown adipocytes is primarily attributed to the mitochondrial inner membrane protein, uncoupling protein-1 (UCP1), which catalyzes a proton leak across the inner mitochondrial membrane, thus uncouples oxidative phosphorylation from ATP synthesis, and converts chemical energy to heat (20, 21). Brown adipocytes are located in the well-defined anatomical BAT depots such as interscapular, peri-aortic, intercostal and mediastinal regions of rodents. In addition to the classical brown adipocytes, beige adipocytes also contribute to thermogenesis. Although beige adipocytes share similar morphological characteristics and thermogenic capacity with classical brown adipocytes, they arise from different precursor cells (22, 23). In humans, although early studies suggested that BAT is present only in neonates to prevent from hypothermia resulted from high body surface area-to-mass ratio, recent positron emission tomography coupled with computer tomography (PET/CT)-based approaches have identified the existence of metabolically-active BAT in the supraclavicular, ventral cervical and thoracic regions of adults (24–26). While the predominant form of the interscapular BAT in human neonates is classical brown adipocytes, BAT in human adults share more molecular features with beige adipocytes (23). Furthermore, both amount and activity of BAT in adults is negatively correlated with body weight, T2D and cardiovascular events, but positively correlated with energy expenditure (24, 25, 27).

### Cellular Composition of Adipose Tissue

Although adipocytes are the dominant cell type in adipose tissue, there are also non-adipocyte compartment named as stromal vascular fraction (SVF), which include preadipocytes, adipose tissue-derived stem cells (ADSCs), endothelial cells, pericytes, and various immune cells. Preadipocytes can be differentiated into mature adipocytes to regulate adipogenesis and WAT expansion (28). ADSCs undergo self-renewal and are multipotent, with the potential to differentiate into numerous cell types, including adipogenic lineages, endothelial cells, osteoblasts, chondrocytes and myocytes (29). Endothelial cells and pericytes provide vasculature to adipose tissue by forming capillaries (22, 30–33). Presence of immune cells was not realized till discovery of adipose-resident macrophages responsible for producing pro-inflammatory cytokines in obese mice and humans in the early 2000s (34, 35). It is now known that adipose tissue is home to both innate immune cells such as macrophages, neutrophils, eosinophils and dendritic cells and adaptive immune cells, including T cells and B cells, which collaboratively play important roles in clearance of apoptotic cells, maintenance of adipose tissue function and homeostasis (36).

### Adipose Tissue Inflammation as a Culprit in Obesity-Related Disorders

A growing body of evidence suggests that chronic inflammation in adipose tissue, characterized by infiltration of pro-inflammatory immune cells and aberrant production of adipokines, is a major contributor to obesity-induced systemic inflammation, insulin resistance and metabolic dysregulation (37, 38). Obesity leads to an expansion of adipose tissue driven by adipocyte hyperplasia and hypertrophy. The lipid-laden adipocytes in obesity undergo necrosis and/or apoptosis, leading to aberrant production of adipokines and altered cellular composition in adipose tissue (39). In obesity, the hypertrophic adipocytes exhibit impaired secretion of anti-inflammatory adipokines such as adiponectin, but augmented secretion of a large number of pro-inflammatory mediators, such as IL-6, C-C motif chemokine ligand 2 (CCL2), IL-1β and resistin that lead to a chronic inflammatory state linking obesity to its cardiometabolic comorbidities including insulin resistance, T2D and cardiovascular events (40).

During the progression of obesity, expansion of adipose tissue also causes infiltration and activation of immune cells involved in both innate and adaptive immunity, which in turn trigger a series of inflammatory responses within the tissue. Among adipose-resident immune cells, macrophages are the most abundant cell type, accounting for 40–50% of total cells of adipose tissue in obese humans (41). In obese adipose tissues, macrophages form crown-like structures (CLSs) surrounding dying or dead adipocytes. The number of adipose tissue–resident macrophages (ATMs) is closely associated with the magnitude of insulin resistance and metabolic perturbance, whereas selective depletion of ATMs by genetic or pharmacological approaches is sufficient to prevent obesity-related insulin resistance and metabolic complications in obese mice (34, 42). Macrophages are highly plastic in nature, exhibiting different phenotypes ranging from the classically activated, pro-inflammatory M1 to alternatively activated, anti-inflammatory M2 in response to changing environment (41). The lean adipose tissue is dominated by M2 macrophages which plays an important role in maintaining the tissue homeostasis through phagocytosis of dead adipocytes, secretion of anti-inflammatory cytokines and other regulatory factors for angiogenesis, adipogenesis, and regulation of adaptive thermogenesis (43). However, obesity causes a striking phenotypic change of ATMs from the anti-inflammatory M2 toward the pro-inflammatory M1, the latter of which produce pro-inflammatory cytokines to exacerbate metabolic inflammation and insulin resistance (41, 43). However, the precise mechanisms whereby adipocyte and various immune cells crosstalk with each other to aggravate obesity-induced adipose inflammation and metabolic dysregulation remain poorly defined.

## Cellular Origin of ADEVs and Their Roles in Cell-Cell Communications

EVs are enclosed by a lipid bilayer and classified into three main classes, including exosomes, MVs and apoptotic bodies (44). Exosomes are a homogenous population of EVs at 30–100 nm in diameter. Biogenesis of exosomes begins from endocytosis-mediated invagination of the plasma membrane, resulting in endocytotic vesicles, which are subsequently transported to the early endosomes. Membranes of the endosomes are budded into the lumen to form intraluminal vesicles (ILVs) or multivesicular bodies (MVBs). MVBs can fuse with lysosomes for degradation or with the plasma membrane to release the internal vesicles into extracellular space as exosomes (45). Exosomes show the same orientation with the plasma membrane composed of a lipid bilayer with extracellular domains of proteins exposed at the surface. The lipid bilayer of exosomes encloses a droplet of cytoplasm containing various types of molecules including nucleic acids, proteins and lipids (45). Cells can also produce MVs with heterogenous populations ranging from 100 to 1,000 nm in diameter. In contrast to exosomes derived from the endolysosomal pathway, MVs are formed by direct budding and shedding from the plasma membrane (44). EVs released from cells undergoing apoptosis are referred to as apoptotic bodies with a diameter of 1,000–5,000 nm (44).

**Figure 1:**
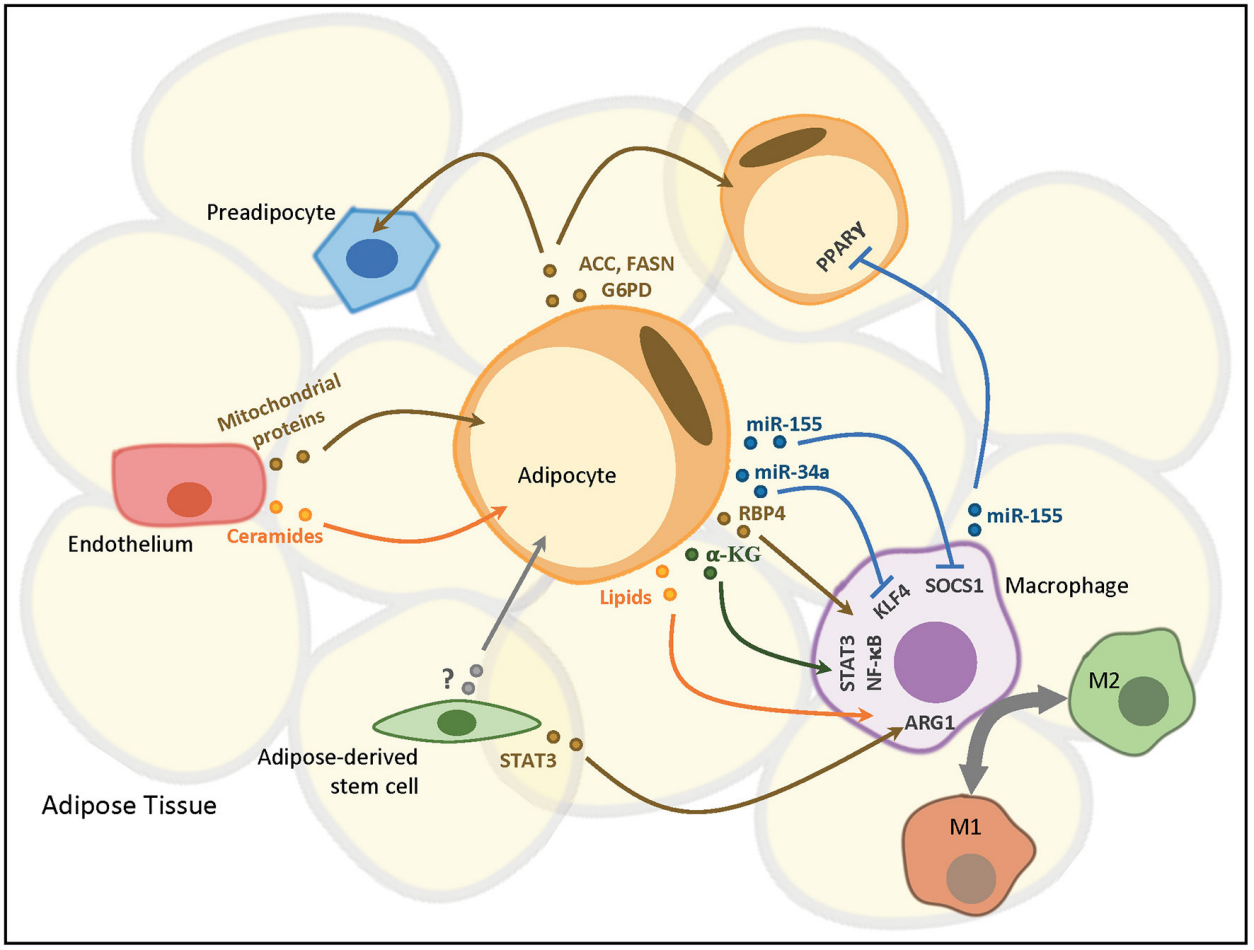
ADEVs-mediated intercellular communication in adipose tissue. Adipocytes mediate the polarization and immunomodulatory responses of adipose-resident macrophages (ATMs) in a paracrine manner via various vesicular components. ATMs reciprocally regulate adipocyte insulin sensitivity by releasing miRNA-containing EVs to adipocytes. Adipocytes also delivery exosomal proteins to neighboring preadipocytes and adipocytes in both a paracrine and an autocrine manner, respectively, to modulate lipogenesis. Adipose-derived stem cells (ADSCs) confer EV-mediated paracrine effects on both adipocytes and ATMs to regulate adipocyte reprogramming and macrophage polarization, respectively. Endothelial cells in adipose tissue transfer EVs containing proteins and lipids capable of modulating cellular signaling pathways to adipocytes. ACC, acetyl-CoA carboxylase; FASN, fatty acid synthase; G6PD, glucose-6-phosphate dehydrogenase; PPARγ, peroxisome proliferator activated receptor gamma; RBP4, retinol binding protein 4; α-KG, α-ketoglutarate; SOCS1, suppressor of cytokine signaling 1; KLF4, krüppel-like factor 4; STAT3, signal transducer and activator of transcription 3; NF-κB, nuclear factor kappa B; ARG1, arginase 1.

Multiple types of cells in adipose tissue, including adipocytes, macrophages, ADSCs and endothelial cells are known to secrete EVs, which in turn, act in a paracrine or endocrine manner to mediate intercellular and inter-organ crosstalk in modulation of adipose tissue and systemic homeostasis (Figure 1), as detailed below.

### Adipocytes

The presence of EVs in the culture medium of adipose tissue explants has been demonstrated in both mouse and human studies (46, 47). Further analysis of EVs isolated from *ex vivo* human adipose tissue explant cultures has identified both adiponectin-positive and adiponectin-negative subsets by differential ultracentrifugation combined with immunoblotting analysis (47). Since adiponectin is expressed predominantly in adipocytes, the adiponectin-positive EVs were suggested to be derived from adipocytes. In agreement with this notion, certain portion of exosomes isolated from mouse serum has also been demonstrated to contain adiponectin and low level of resistin (48). However, as detection of adiponectin in EVs was achieved by immunoblotting, whether adiponectin is located on the surface or inside of adipocyte-derived EVs remains unknown. The secretion of EVs from adipocytes has been further confirmed by *in-vitro* cultures of rat primary adipocytes or adipocytes differentiated from mouse 3T3-L1 pre-adipocytes and human Simpson Golabi Behmel Syndrome (SGBS) pre-adipocytes (47, 49). In addition to adiponectin and resistin, there are several other adipocyte-specific proteins have been identified as the markers of adipocyte-derived EVs, including perilipin A and fatty acid binding protein 4 (FABP4) (50, 51).

Although adipocyte-derived exosomes account for a minority of circulating exosomes under normal condition (52), production of ADEVs can vary under different conditions. For example, the circulating level of lipid-filled vesicles derived from adipocytes was increased by approximately 2-folds in obese mice vs. lean animal (52). Similarly, the number of exosomes isolated from VAT was elevated in human patients with insulin resistance (47). Under chronic cold exposure, the number of exosomes released from explants isolated from both interscapular BAT and inguinal WAT of mice was significantly induced. *In-vitro* studies showed that release of exosomes from beige and brown adipocytes was increased by treatment with cAMP, which is the second messenger induced by cold exposure or beta-adrenergic stimulation (53).

Adipocyte-derived EVs confer in part the paracrine interaction between adipocytes and macrophages. EVs released from human adipocyte culture were able to induce differentiation of monocytes into ATM-like macrophages *in vitro*. Adiponectin-positive EVs from human adipose tissue explants were more potent than the adiponectin-negative subset in promoting monocyte differentiation into ATMs as they induced the expression of mixed pro- and anti-inflammatory markers, which are characteristic of ATMs, in monocytes *in vitro* (47). Furthermore, adipocyte-derived EVs isolated from high-fat diet (HFD)-fed mice drove polarization of macrophages toward the pro-inflammatory M1 phenotypes in bone marrow-derived macrophages (BMDMs) *in vitro* by miR-155, which inhibited the expression of suppressor of cytokine signaling 1 (SOCS1), leading to suppression of signal transducer and activator of transcription 6 (STAT6) (54). In addition to adipose tissue, adipocytes also exist in tumor microenvironment, including melanoma. It is reported by recent studies that adipocyte-secreted exosomes were taken up by tumor cells, resulting in increased melanoma migration and invasion through fatty acid oxidation. Such effects were amplified in obese animals (55, 56).

### Macrophages

In addition to adipocytes, ATMs also produce EVs to modulate inflammatory responses and metabolic homeostasis. *In vitro*, secretion of EVs was detected in the culture medium of human THP-1-derived macrophages (14, 57). These EVs were identified as exosomes with a diameter of 30–100 nm by using transmission electron microscopy (14). The macrophage-derived exosomes can be effectively internalized into adipocytes. Exosomes from LPS-activated macrophages promote the expression of inflammation-related genes in adipocytes (57). Interestingly, when THP-1 monocytes-derived macrophages are polarized to M1 or M2 phenotype by LPS plus IFN-γ or IL-4, respectively, exosomes derived from M1 macrophages impair insulin signaling in human adipocytes, while the M2 macrophage-derived exosomes enhance insulin signaling and glucose uptake in adipocytes (14). Likewise, EVs harvested from ATMs isolated from VAT of mice were also found to be exosomes as they are 30–100 nm in size (13). It is further evidenced by detection of exosomal membrane markers in the EVs, including TSG101, syntenin 1, CD63, and CD9. In line with the *in-vitro*-based findings, treatment with ATM-derived exosomes from lean mice ameliorated diet-induced glucose intolerance and insulin resistance in obese mice, whereas administration of exosomes isolated from ATMs of obese mice promoted glucose intolerance and insulin resistance in lean recipients (13). These studies collectively support a critical role of ATM-derived exosomes in the regulation of neighboring adipocytes under physiological and pathological conditions.

### Adipose-Derived Stem Cells (ADSCs)

ADSCs have emerged as a potential tool for regenerative therapy due to its multipotency in differentiating into different types of cells (58). Additionally, ADSCs are also a critical player in immune regulation, and have shown potential for treatment of inflammatory and autoimmune diseases, including colitis, autoimmune diabetes and arthritis, as well as to resolve obesity-induced inflammation and metabolic dysregulation by polarization of macrophages toward the anti-inflammatory M2 phenotypes (59–62). These beneficial effects may be attributed at least in part to the paracrine effects of EVs produced from ADSCs. Zhao *et al*. isolated ADSCs from mouse VAT and found that ADSC-derived EVs were approximately 100 nm in diameter and positive for the exosomal markers TGS101, CD9, CD63, HSP90, and ALIX, thus of exosomal origin (15). It has been shown in another study that human primary ADSCs also secreted 40–100 nm particles, which had the typical characteristics of exosomes (63). However, Katsuda et al. reported that human ADSC-derived exosomes had a peak size distribution of 150–200 nm which was larger than that reported by others. However, exosomal markers CD63 and HSP90 were present, suggesting that the size range of exosomes may differ among different cell types (64).

ADSC-derived exosomes isolated from patients with and without cancer show distinct miRNA profiles. Selective enrichment of certain miRNAs, including let-7-a-1, miR-21, and miR-1260b has been identified in ADSC-derived exosomes from cancer patients (65). Treatment of hepatocellular carcinoma cells with ADSC-exosomes containing miR-122 showed increased sensitivity to chemotherapies (66). Human ADSC-derived exosomes promoted migration of breast cancer cell line (63). ADSC-derived exosomes can be internalized into ATMs, and treatment of obese mice with ADSC-derived exosomes isolated from mouse VAT attenuated obesity and insulin resistance by inducing polarization of macrophages toward the M2 phenotypes through transactivation of arginase-1 by exosome-carried active STAT3, thus beiging of WAT (15). In addition to the undifferentiated ADSCs, EVs isolated from human ADSCs during white and beige adipogenic differentiation provided biochemical cues such as miRNAs to induce the differentiation of ADSCs into white and beige adipocytes, thereby promoting adipogenesis and adipose tissue remodeling, respectively (67).

### Endothelial Cells

A recent study also identified adipose tissue endothelial cells as a source of ADEVs (68). These ADEVs are enriched with the exosomal markers CD9, CD63, TSG101, and ALIX. Production of EVs from adipose endothelial cells was increased under the fasted condition, mainly through the action of glucagon. As endothelial cells are located at the interface between the circulation and adipose tissue extracellular space, endothelial cell-derived ADEVs can take up proteins and lipids such as mitochondrial components and ceramides from the bloodstream, and subsequently release the components to the adjacent adipocytes through internalization (68). Notably, the changes of EV secretion from adipose endothelial cells in response to fasting and refeeding was absent in dietary or genetic obese mouse models, implicating the possible involvement of dysregulated adipose endothelial cell-derived EVs in the pathogenesis of obesity and its related metabolic diseases.

## Major Components of ADEVs and Their Roles in Immune Responses and Metabolic Regulation

**Figure 2:**
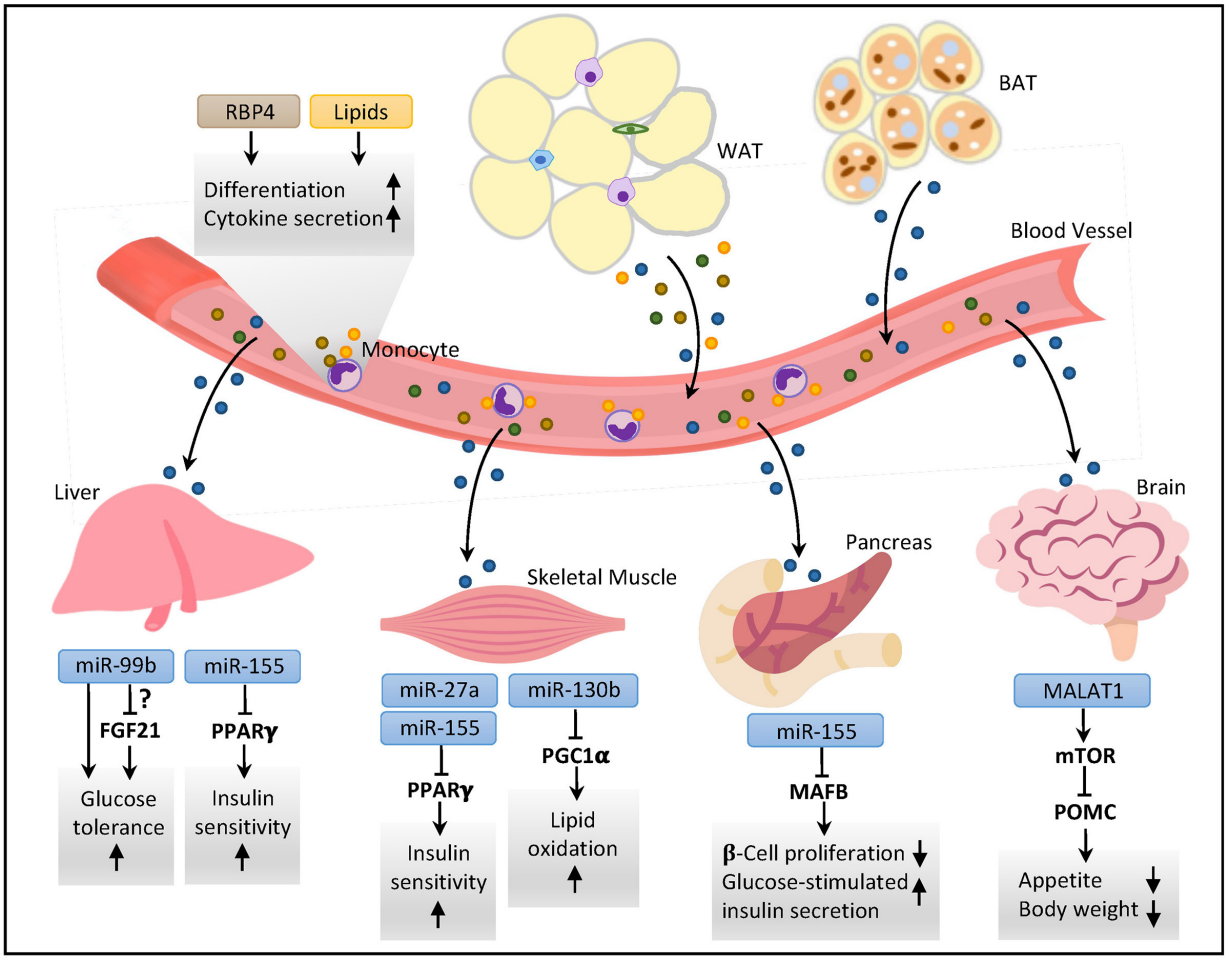
ADEVs-mediated interorgan crosstalk in metabolic regulation. Both white and brown adipose tissues secrete EVs containing various types of vesicular components into the circulation. The ADEVs can act as endocrine factors affecting metabolic profiles in distal organs by sending bioactive vesicular molecules. In the liver, exosomal miRNAs modulate glucose tolerance and insulin sensitivity through modulation of peroxisome proliferator activated receptor gamma (PPARγ) and perhaps fibroblast growth factor 21 (FGF21). In skeletal muscle, miRNAs regulate insulin sensitivity and lipid oxidative capacity through PPARγ and PPARγ coactivator 1α (PGC1α), respectively. In pancreas, ADEV-derived miRNAs modulate β-cell mass and insulin secretion. In brain, ADEVs-derived long non-coding RNA metastasis-associated lung adenocarcinoma transcript-1 (MALAT1) regulates mTOR signaling in hypothalamic pro-opiomelanocortin (POMC) neurons to control appetite and body weight. In the bloodstream, exosomal proteins and lipids affect the differentiation and immunomodulatory responses of monocytes. RBP4, retinol binding protein 4; WAT, white adipose tissue; BAT, brown adipose tissue; MAFB, v-maf musculoaponeurotic fibrosarcoma oncogene family protein B.

EVs exert their biological functions by carrying various types of bioactive cargos including mRNAs, miRNAs, DNA, proteins and lipids to their target cells through phagocytosis or endocytosis (55, 69, 70), which in turn mediate cell-cell communications. Additionally, since EVs have the same transmembrane proteins on their surface as the cell of origin, they can also act as the ligands directly binding and activating the surface receptor of target cells to initiate cellular signaling (71). Likewise, ADEVs modulate immune responses in local adipose tissues through cell-cell communication, and systemic insulin sensitivity, glucose and lipid metabolisms through their distal effects on other major metabolic organs such as liver, skeletal muscle, and brain (Figure 2). Such local and distal effects of ADEVs are attributed to their unique vesicular composition, which has been characterized in great details (Table 1).

**Table 1:** Summary of major ADEV cargos and their functions in immune responses and metabolic regulation.

**Cargo**	**Donor**	**Recipient**	**Molecular Target**	**Functions**	**References**
**miRNAs**					
miR-99b	Adipocytes	Hepatocytes	FGF21	Glucose intolerance **↓**	(12)
miR-155	Adipocytes	Macrophages	SOCS1	M1 polarization of macrophages **↑**, adipocyte insulin signaling **↓**	(54)
miR-34a	Adipocytes	ATMs	KLF4	M2 polarization of macrophages **↓**, adipose inflammation **↑**	(11)
miR-130b	Adipocytes	Myocytes	PGC1α	Lipid oxidation ↓	(72)
miR-27a	Adipocytes	Myocytes	PPARγ	Insulin resistance **↑**	(73)
miR-155	ATMs	Adipocytes, hepatocytes, myocytes,β cells	PPARγ MAFB	Insulin resistance **↑**β cells proliferation ↑, glucose-stimulated insulin secretion ↓	(13, 74)
**Proteins**					
ACC, FASN, G6PD	Adipocytes	Adipocytes, preadipocytes	*De novo* lipogenesis	Adipogenesis and lipogenesis **↑**	(75)
RBP4	Adipocytes	Macrophages	TLR4 signaling	Macrophage activation **↑**, adipose inflammation **↑**	(46)
STAT3	ADSCs	ATMs	Arginase-1	M2 polarization of macrophages **↑**, adipose inflammation **↓**, beiging of WAT **↑**	(15)
Mitochondrial components	Adipose endothelial cells	Adipocytes	Mitochondrial respiratory chain, lipid metabolism	Respond to changes in systemic nutrient status	(68)
**Lipids and others**					
Neutral lipids	Adipocytes	Monocytes, ATMs	Lysosomal catabolism	Lipid release, monocyte differentiation to ATM	(52)
Ceramides	Adipose endothelial cells	Adipocytes	Stress-related signaling pathways	Involved in pathological signaling in T2D	(68)
α-KG	Adipocytes	Macrophages	STAT3/NF-κB signaling	M2 polarization of macrophages **↑**	(76)

*FGF21, fibroblast growth factor 21; SOCS1, suppressor of cytokine signaling 1; ATMs, adipose tissue-resident macrophages; KLF4, krüppel-like factor 4; PPARγ, peroxisome proliferator activated receptor gamma; PGC1α, PPARγ coactivator 1α; MAFB, v-maf musculoaponeurotic fibrosarcoma oncogene family protein B; ACC, acetyl-CoA carboxylase; FASN, fatty acid synthase; G6PD, glucose-6-phosphate dehydrogenase; RBP4, retinol binding protein 4; TLR4, toll-like receptor 4; STAT3, signal transducer and activator of transcription 3; ADSCs, adipose-derived stem cells; WAT, white adipose tissue; T2D, type 2 diabetes; α-KG, α-ketoglutarate; NF-κB, nuclear factor kappa B*.

### miRNA, mRNA and lncRNA

The exosome-mediated cellular signaling is largely dependent on their composition of miRNAs, which are small non-coding RNA molecules that post-transcriptionally regulate gene expression by binding to the 3′-untranscribed region of target mRNAs, leading to mRNA degradation and repression of translation. miRNAs are critically involved in adipogenesis and regulation of adipose tissue functions (77). Recently, ADEVs have been found as an important source of circulating miRNAs in both mice and humans. It was evidenced by a significant reduction in exosomal miRNAs in serum of adipocyte-specific Dicer knockout (ADicerKO) mice, which have abrogated miRNA processing in adipocytes (12). DICER is a key enzyme that cleaves pre-miRNAs into mature miRNA, thus important for miRNA biogenesis (78). The same study also examined the circulating exosomal miRNA profiles in patients with congenital generalized lipodystrophy and patients with HIV-associated lipodystrophy who have general loss of adipose tissue and reduced expression of adipose Dicer respectively, and found that dominant miRNAs in exosomes were significantly downregulated in the serum of both patient cohorts, suggesting that circulating miRNAs in humans also originate mainly from ADEVs (12). Defects in ADEV-derived miRNA production resulted in reduced WAT, whitening of BAT, insulin resistance and dyslipidaemia in the ADicerKO mice, demonstrating the importance of adipose tissue-specific exosomal miRNAs in the physiological regulation of systemic energy metabolism. Transplantation of BAT, but not WAT from wild-type mice in ADicerKO mice improved glucose tolerance and insulin resistance in the recipient mice which was associated with reduced production and secretion of FGF21 from the liver (12). Further investigation revealed that mRNA expression of Fgf21 in hepatocytes was suppressed by ADEV-derived miR-99b from BAT, suggesting that BAT-derived exosomal miRNAs mediate the adipose-liver crosstalk to modulate glucose homeostasis. However, both animal and clinical studies have shown beneficial effects of FGF21 in improving insulin sensitivity and alleviating hyperglycemia (79). Therefore, it is unlikely that the metabolic benefits of BAT-derived ADEVs are attributed to reduction in hepatic FGF21 expression. Further studies are needed to investigate the detailed molecular mechanism underlying the effects of BAT-derived exosomal miRNAs in the regulation of systemic glucose homeostasis.

ADEVs derived from different adipose depots appear to contain distinct miRNA composition. For example, miR-34a is selectively enriched in the exosomes from VAT, but not in SAT in both rodents and humans (11). Furthermore, high fat diet feeding leads to a progressive increase of miR-34a in the exosomes isolated from adipocytes in VAT in mice. The adipocyte-derived miR-34a is transported to the adjacent macrophages by exosomal delivery and drives the polarization of macrophages toward the pro-inflammatory M1 phenotypes by suppression of transcription factor Krüppel-like factor 4 (KLF4), which is important in maintenance of M2 macrophage phenotypes. Conversely, adipocyte-selective ablation of miR-34a protects mice against obesity-induced adipose inflammation, systemic insulin resistance and NAFLD (11). Selective enrichment of miR-34a in VAT may explain why this adipose depot is more susceptible to inflammation and is more harmful to cardiometabolic health than SAT. In addition, miRNA-containing exosomes released from ATMs can modulate systemic insulin resistance. Administration of obese ATM-derived exosomes in lean mice impaired insulin sensitivity and glucose tolerance (13, 80). Uptake of ATM-exosomes can be detected in the liver, muscle and adipose tissues of mice. *In-vitro* experiments showed that exosomes derived from obese ATMs directly impaired insulin signaling in adipocytes, myocytes and hepatocytes (80). These effects were possibly attributed to obesity-induced changes in miRNA contents in the ATM-exosomes, such as miR-155, which target the nuclear receptor PPARγ (13). Likewise, the distal effects of the exosomal miR-27a released from adipocytes of obese mice on induction of insulin resistance in skeletal muscle were also attributed to its repression of PPARγ (73). Adipocytes also regulate lipid catabolism in skeletal muscle via exosomal miR-130b. miR-130b has been shown to inhibit the expression of PPARγ coactivator 1α (PGC1α), which is important in lipid oxidative capacity and mitochondrial function (72). In addition to the aforementioned roles of miR-155 on adipocytes, hepatocytes and myocytes, exosomal miR-155 derived from ATMs of obese mice also exerts profound regulation on pancreatic β cells, leading to impaired insulin secretion and increased β cell proliferation by repressing the expression of v-maf musculoaponeurotic fibrosarcoma oncogene family protein B (MAFB) (74).

There is emerging evidence showing that environmental changes can alter the composition of adipose-derived exosomal miRNA, which in turn participates in adaptive responses to metabolic stresses. In high-attitude population, hypoxia and cold temperature causes downregulation of exosomal miR-210/92a from WAT, thereby increasing the thermogenic activity of BAT possibly by upregulation of FGFR1 (81). ADEVs may participate in the regulation of the inflammasome activation. EVs derived from both ADSCs and epidural fat-mesenchymal stem cells inhibit Nod-like receptor pyrin domain-containing three (NLRP3) inflammasome activation (82, 83). MiR-223, possibly of ADEV origin, is reduced in blood from patients with T2D and obesity (84). This reduction in miR-223 is believed to contribute to the increased adipose tissue inflammation in obesity as miR-223 can inhibit inflammation by targeting NLRP3, which is a key component of the inflammasome (85). However, it is currently unclear how adipose tissues sense the environmental and nutritional changes to alter the vesicular composition of miRNAs under different pathophysiological conditions.

In addition to miRNAs, mRNAs have been found to be present in EVs. Valadi and colleagues provided the first evidence demonstrating that EVs secreted from mast cells contained substantial amount of mRNAs, which were functional as they can be transferred to other cells and translated into new proteins in the recipient cells (70). Subsequent analysis of EVs derived from adipocytes differentiated from mouse 3T3-L1 cell line revealed that ADEVs also contained mRNAs encoding genes involved in metabolic and inflammatory processes (86). In contrast, exosomal mRNAs were not detected in VAT or SAT from either lean or obese human subjects (87). The discrepancy may be resulted from the difference in cellular sources of exosomes and possible difference in miRNA species that might mediate intrinsic degradation of mRNAs in ADEVs from human subjects.

Long non-coding RNAs (lncRNAs) have been emerged as critical regulators to control the development and functions of various metabolic tissues. For example, brown adipose tissue-specific lncRNA 1 (lnc-BATE1) was induced during brown adipocyte differentiation and enriched in BAT compared to WAT in mice. siRNA-mediated knockdown of lnc-BATE1 impaired differentiation of brown adipocytes *in vitro* (88). Liver-specific triglyceride regulator lncRNA (lnc-LSTR) was identified as an important regulator of hepatic lipid metabolism. Knockdown of lnc-LSTR in mice lowered serum triglyceride levels by induction of apolipoprotein C2 (ApoC2), which promotes lipoprotein lipase-mediated hydrolysis of triglyceride-rich lipoproteins (89). Recently, it has been reported that lncRNAs are transferred by ADEVs to mediate the interconnection between adipose tissue and the central nervous system. In particular, adipocyte-derived exosomal metastasis-associated lung adenocarcinoma transcript-1 lncRNA (lnc-MALAT1), which is elevated in obese mice, has been shown to target hypothalamic pro-opiomelanocortin (POMC) neurons to upregulate mTOR and thereby downregulate POMC expression, resulting in increased appetite and weight gain in lean mice (90).

### Proteins

The nature of proteins released from adipocyte-derived exosomes has been characterized by proteomic profiling of exosomes produced by human primary adipocytes in comparison with the overall secretome of the same cells (69). This analysis identified 884 proteins, called as exoadipokines. Among them, 212 proteins commonly found in both secretome of human adipose tissues and exosomes are mainly involved in inflammation and fibrosis, whereas 672 proteins specific for exosomes which are assigned to signaling pathways and membrane-mediated process (69). Notably, exosomes were found to be enriched in proteins without classical signaling peptides that direct proteins to the traditional secretory pathway, suggesting a significant contribution of exosomes to the overall human adipokinome (69). Notably, different proteomic profiles in adipocyte-derived EVs have been observed between obese diabetic rats and obese non-diabetic counterparts (91). Exosomes derived from obese diabetic mice are enriched in proteins and enzymes involved in lipolysis and glycerol export, which may explain ectopic lipid accumulation in major metabolic organs and hence systemic insulin resistance (91). Intriguingly, hypoxia, which is present in obese adipose tissue, has also been shown to alter proteomic composition of ADEVs *in vitro* (75). In particular, enzymes involved in *de novo* lipogenesis such as acetyl-CoA carboxylase (ACC), fatty acid synthase (FASN) and glucose-6-phosphate dehydrogenase (G6PD) were selectively enriched in exosomes derived from adipocytes under the hypoxic condition, which may increase lipid accumulation in recipient adipocytes and preadipocytes.

Exosomal proteins in ADEVs are functionally involved in the paracrine crosstalk between adipocytes and macrophages in adipose tissue. Adiponectin-positive EVs derived from adipocytes can promote differentiation of monocytes into ATMs, which are associated with the production of immunomodulatory proteins such as TNFα, macrophage-colony-stimulating factor (MCSF) and retinol binding protein 4 (RBP4) (47). The functional relevance of exosomal protein RBP4 in monocyte differentiation has been substantiated in another independent study showing that ADEVs from *ob/ob* mice contained a higher level of RBP4, and exosomal RBP4 induced macrophage activation and production of pro-inflammatory cytokines *in vitro* (46). Additionally, exosomes from ADSCs contain active STAT3, which can be transported to macrophages to induce polarization of macrophages toward the anti-inflammatory M2 phenotypes through transcriptional activation of arginase-1 (15). Treatment of obese mice with the ADSC-derived exosomes alleviated diet-induced insulin resistance and glucose intolerance by reducing adipose inflammation and enhancing beiging of WAT (15). These studies collectively support the immunomodulatory effects of exosomal proteins, in reminiscence of classical adipokines and chemokines, in the interconnection between obesity and inflammation. However, loss-of-function studies are warranted to confirm the requirement of individual exosomal proteins in the regulation of adipose immune responses and insulin sensitivity.

### Lipids and Other Cargos

In addition to aforementioned miRNAs, mRNAs and proteins, lipids and other cargos also act as the signaling molecules conferring the effects of ADEVs. A recent study identified lipid-filled exosomes released from adipocytes, and these lipid-enriched exosomes play an important role in transporting lipids from adipocytes to macrophages (52). Furthermore, these lipid-filled exosomes are also sufficient to induce differentiation of bone marrow-derived monocytes into ATM-like macrophages *in vitro* (52). The number of the lipid-filled exosomes secreted from adipocytes was more than doubled in obese mice relative to the lean mice which might be an additional mechanism for obesity-associated adipose inflammation. This study also proposed these lipid-filled exosomes as a new pathway of lipid release from adipocytes independent of the canonical lipolysis. However, little is known on how different types of bioactive lipids are selectively enriched in ADEVs and exert their local and/or distal effects on immunological and metabolic regulation. Adipocytes are also found to produce exosomes containing α-ketoglutarate. Melatonin, a hormone released from the pineal gland with anti-inflammatory activities, promoted the secretion of exosomes containing α-ketoglutarate from adipocytes, whereas uptake of exosomal α-ketoglutarate by macrophages facilitated the polarization toward the anti-inflammatory M2 phenotype and thus alleviated adipose inflammation in obesity (76).

## Clinical Implications of ADEVs in Metabolic Diseases

Measurement of ADEVs in adipose tissue is minimally invasive and can be potentially used as an alternative approach to evaluate metabolic health. Changes in circulating EVs have been associated with various metabolic diseases, including obesity, T2D and NAFLD, making them attractive biomarkers for diagnosis and risk prediction of these diseases (92). However, the extent to which circulating EVs are contributed by ADEVs is currently unclear. Several adipose markers, including adiponectin, FABP4 and perilipin A have been used to identify EVs released from adipose tissues (50, 51). The level of circulating EVs positive for perilipin A and thus of adipocyte origin was dramatically increased in both mice with diet-induced obesity and obese human patients (50). Furthermore, in obese humans, the circulating level of the EVs enriched with perilipin A was positively correlated with plasma insulin level and homeostatic model assessment of insulin resistance (HOMA-IR), supporting potential use of perilipin A-positive EVs as the biomarker of insulin resistance (50).

Metabolic status can be also reflected by changes in vesicular miRNAs in ADEVs. By using FABP4 as a marker to identify ADEVs from the circulation, Hubal et al. found that the miRNA content of circulating ADEVs targeting various genes in the canonical insulin receptor-mediated signaling pathway was significantly altered 1 year-after gastric bypass bariatric surgery, and the changes were closely associated with improvements in insulin sensitivity, suggesting that FABP4-positive ADEVs might be useful to monitor the response of obese patients to the intervention with bariatric surgery (51). However, as FABP4 is also expressed in several types of immune cells and endothelial cells, ADEVs may only account for a proportion of the total FABP4 positive EVs. In addition, the circulating level of exosomal miR-92a possibly of BAT origin was found to be negatively associated with BAT activity as measured by ^18^F-fluorodeoxyglucose PET/CT in two human cohorts, and thus may represent a potential biomarker for monitoring BAT activity in humans, which is much more cost-effective than PET/CT-based approaches (53).

ADEVs from different adipose depots are differentially associated with metabolic health and disease. The number of EVs derived from omental adipose tissue, but not SAT, correlated positively with HOMA-IR in overweight patients (47). It has also been reported that the abundance of EVs in VAT was positively correlated with serum levels of alanine aminotransferase (ALT) and aspartate aminotransferase (AST), which are the well-established markers for liver injury, whereas the number of EVs in SAT was inversely associated with waist circumference and metabolic syndrome (93). These clinical observations suggest that differential effects of SAT and VAT on metabolic health may be related to the distinct amount and/or composition of EVs from these two adipose depots.

## Therapeutic Potential of ADEVs for Metabolic Diseases

Owing to the easy accessibility from the bloodstream, the ability to transport the bioactive cargos and surmount biological barriers, the possibilities to modify the content with bioengineering, and target specificity, EVs have been emerged as a cell-free therapy for treatment of various diseases (94). In particular, EVs from mesenchymal stem cells (MSCs) could fully mimic the immunomodulatory and regenerative functions of parental MSCs, and have therefore been exploited as potential therapeutic agents for various inflammatory diseases and regenerative medicine targeting lung, liver, bone, kidney, brain and heart (95), and a large number of clinical trials on MSC-derived EVs have been publicly registered in recent years.

Although ADSCs have been shown to possess promising therapeutic efficacy for Crohn's disease, idiopathic pulmonary fibrosis and chronic kidney diseases (NCT03939741) in various clinical studies, the therapeutic application of ADSC-derived EVs are still at the early stage (96, 97). Nevertheless, there is a growing number of preclinical studies suggesting that ADEVs have the great therapeutic potential for obesity-related metabolic diseases. Treatment of obese mice with ADSC-derived exosomes obtained from lean mice caused a significant reduction of adipose inflammation and beiging of WAT, thereby leading to obvious metabolic improvements, including weight loss, alleviation of insulin resistance and hepatic steatosis (15). Administration of exosomes isolated from BAT or serum of wild-type mice significantly improved insulin sensitivity and normalized serum lipids in ADicerKO mice (12). Likewise, treatment of mice with EVs isolated from human ADSCs during adipogenic differentiation to beige adipocytes attenuated diet-induced obesity and hepatic steatosis (67). By contrast, infusion of mice with EVs isolated from human ADSCs during adipogenic induction to white adipocytes promoted adipogenesis and expansion of WAT, suggesting the therapeutic potential for lipodystrophy, a disorder associated with reduced number of circulating exosomes (12).

In addition to use endogenous ADEVs as a therapeutic agent, bioengineering ADEVs by modifying the bioactive cargos may represent another viable approach to develop effective treatment for obesity-related metabolic complications. An example is to deplete those miRNAs causally involved in metabolic inflammation and insulin resistance. In this connection, genetic ablation of exosomal miR-34a, which is highly enriched in exosomes secreted from VAT and contributes to adipose inflammation by inducing M1 macrophage polarization, has been shown to reverse obesity-induced insulin resistance, glucose intolerance and fatty liver in mice (11). Similarly, treatment of dietary obese mice with antisense RNA for miR-34a restores hepatic β-klotho expression and FGF19 signaling, leading to attenuation of fatty liver disease (98). It is also possible to exogenously load ADEVs with transcriptional factors participating in M2 macrophage polarization (15), thereby reducing obesity-related metabolic diseases.

## Conclusions and Future Perspectives

Emerging evidence from both *in-vitro* and *in-vivo* studies support the role of ADEVs as important players mediating cell-cell communication within adipose tissues as well as interorgan crosstalk between adipose tissue and other distal organs, thus participating in the regulation of local immune responses, tissue remodeling, systemic insulin sensitivity, and energy homeostasis (Figures 1, 2). Aberrant production and/or function of ADEVs are implicated in the pathogenesis of obesity and its related metabolic complications. ADEVs are heterogeneous in terms of size, composition and origin, with ADEVs derived from different adipose depots exhibiting distinct or even opposite functions. However, we are still in the early stage in understanding biogenesis, regulation and pathophysiological functions of ADEVs, and there are many important questions which remain to be addressed: How is the cargo composition of ADEVs regulated? How does obesity cause dysregulation in the number and cargo composition of ADEVs? What determines the target specificity of ADEVs? How do different cargos affect the functions of target cells? Furthermore, the clinical investigation of ADEVs as diagnostic biomarkers and therapeutics for metabolic diseases are constrained by several technical difficulties: First, there is no well-established, definitive marker(s) for ADEVs, and it is therefore difficult to dissect the contribution of ADEVs to circulating EVs, and to precisely measure the changes of circulating ADEVs in different metabolic diseases. Second, it is difficult to isolate ADEVs with high purity using current experimental approaches, and contamination with other particles such as lipoproteins remains a major concern. Moreover, due to the heterogeneity of ADEVs, a mixture of EV populations with different cargos exists in the same cell, causing the difficulties in obtaining a subtype with a specific set of cargos for functional characterization. Further technological advances in molecular and functional characterization of ADEVs will help to enhance our knowledge in metabolic regulation and to facilitate the development of novel therapeutics for treatment of obesity and its related metabolic complications.

## Author Contributions

ZH designed the review, collected references, prepared the table and figures, and wrote the manuscript. AX conceptualized the idea, provided critical suggestions and edited the manuscript. All authors contributed to the article and approved the submitted version.

## Conflict of Interest

The authors declare that the research was conducted in the absence of any commercial or financial relationships that could be construed as a potential conflict of interest.
